# Understanding community perceptions, social norms and current practice related to respiratory infection in Bangladesh during 2009: a qualitative formative study

**DOI:** 10.1186/1471-2458-11-901

**Published:** 2011-12-04

**Authors:** Fosiul A Nizame, Sharifa Nasreen, Leanne Unicomb, Dorothy Southern, Emily S Gurley, Shaila Arman, Mohammad A Kadir, Eduardo Azziz-Baumgartner, Stephen P Luby, Peter J Winch

**Affiliations:** 1 International Centre for Diarrhoeal Disease Research, Bangladesh (ICDDR, B), Dhaka, Bangladesh; 2 Centers for Disease Control and Prevention (CDC), 1600 Clifton Road, Atlanta, GA, USA; 3 Johns Hopkins Bloomberg School of Public Health, Baltimore, MD, USA

**Keywords:** Non-pharmaceutical intervention, Pandemic, Influenza, Coughing, Sneezing, Respiratory hygiene

## Abstract

**Background:**

Respiratory infections are the leading cause of childhood deaths in Bangladesh. Promoting respiratory hygiene may reduce infection transmission. This formative research explored community perceptions about respiratory infections.

**Methods:**

We conducted 34 in-depth interviews and 16 focus group discussions with community members and school children to explore respiratory hygiene related perceptions, practices, and social norms in an urban and a rural setting. We conducted unstructured observations on respiratory hygiene practices in public markets.

**Results:**

Informants were not familiar with the term "respiratory infection"; most named diseases that had no relation to respiratory dysfunction. Informants reported that their community identified a number of 'good behaviors' related to respiratory hygiene, but they also noted, and we observed, that very few people practiced these. All informants cited hot/cold weather changes or using cold water as causes for catching cold. They associated transmission of respiratory infections with close contact with a sick person's breath, cough droplets, or spit; sharing a sick person's utensils and food. Informants suggested that avoiding such contact was the most effective method to prevent respiratory infection. Although informants perceived that handwashing after coughing or sneezing might prevent illness, they felt this was not typically feasible or practical.

**Conclusion:**

Community perceptions of respiratory infections include both concerns with imbalances between hot and cold, and with person-to-person transmission. Many people were aware of measures that could prevent respiratory infection, but did not practice them. Interventions that leverage community understanding of person-to-person transmission and that encourage the practice of their identified 'good behaviors' related to respiratory hygiene may reduce respiratory disease transmission.

## Background

Many human respiratory pathogens including influenza, respiratory syncytial virus, and the coronavirus that causes severe acute respiratory syndrome (SARS) are spread by coughing or sneezing [1-3]. These respiratory viruses contribute importantly to the global respiratory disease burden [4]. When people are infectious and cough or sneeze, these viruses can be expelled through aerosols and large droplets and spread virus from person to person [2-6].

Transmission prevention is accorded a high priority in Bangladesh for two reasons: the high respiratory disease burden and high population density. Acute respiratory infection is a major cause of child mortality in Bangladesh, accounting for 21% of all deaths in children aged less than 5 years from 2000 to 2004 [7]. When pandemic influenza A (H1N1) occurred in Bangladesh in 2009, there was concern for its potential to rapidly spread due to the high population density and lack of respiratory hygiene. To contain or reduce transmission of viruses such as pandemic influenza the World Health Organization (WHO) recommended non-pharmaceutical interventions and public health messages on maintenance of respiratory hygiene/cough etiquette [8]. The Centers for Disease Control and Prevention (CDC) identified certain respiratory hygiene measures, including: covering the nose and mouth when coughing or sneezing; using tissues to contain respiratory secretions; disposal of used tissues in the nearest waste receptacle; and washing hands with soap after having contact with respiratory secretions and contaminated objects. CDC recommended that if persons do not have a tissue, they should cough or sneeze into their upper sleeve, not into their hands, to stop the spread of germs [1,6]. Nevertheless, a recently conducted quantitative study in a rural and an urban site in Bangladesh found that very few people in these areas currently practice respiratory hygiene. Of the 1,122 observed coughing and sneezing events, 907 (81%) people coughed or sneezed into the air, 119 (11%) into their hands and 83 (7%) into their clothing [9].

To design an effective intervention to improve respiratory hygiene in a community, it is important to understand the community's perceptions on respiratory infections and their perceived barriers to practice respiratory hygiene. Finding ways to communicate health messages that could prevent respiratory infections is difficult for several reasons: respiratory diseases tend to have multiple symptoms rather than a single symptom, e. g. coughing, fever, and difficulty breathing; symptoms of respiratory infections exist on a continuum from mild to severe; people perceive respiratory illnesses as "mild" events even among those who have severe signs and symptoms; some symptoms are related to non-infectious illnesses such as asthma or allergies; some respiratory viruses are spread through the air and thus not easily traced [10-13]. The inability to recognize the severity or understand transmission patterns of respiratory illness is a concern because when viral respiratory infections occurs within a community people may not respond or may take ineffective actions to reduce person-to-person spread of the viruses. For example, they might try to avoid being exposed to cold weather or water rather than avoiding persons with respiratory symptoms. Improved respiratory hygiene, such as coughing or sneezing into the upper sleeve, could reduce the risk of respiratory illness, especially in densely populated countries like Bangladesh, because both crowding and poor ventilation facilitate respiratory infections spread quickly from person to person [3]. In low-income countries like Bangladesh, disposable tissues are considered a luxury and not commonly used.

In this study, we explored whether the determinants of respiratory hygiene in our study population matched our hypothesis. We hypothesized that persons in the community share traditional beliefs, culture, social norms and perceived risk of illness about the transmission of respiratory infections that may not compatible with germ theory. These determinants are important to address in a successful respiratory hygiene intervention. Our formative qualitative research study examined the above hypothesis and this paper reports our findings about community perceptions on respiratory infections, why they occur, how they are spread, and the preventive measures that people take to protect themselves and their families. We collected these data to facilitate the development of appropriate behavior change interventions to interrupt respiratory disease transmission.

## Methods

### Study sites

We conducted the study during December 2008 to September 2009 in an urban area of Mirpur, in the capital city Dhaka, and in rural Fulbaria, a sub-district of Mymensingh District, in north central Bangladesh. We selected these sites as they are typical of urban and rural areas in Bangladesh and both had other ongoing International Centre for Diarrhoeal Disease Research, Bangladesh (ICDDR, B) studies that allowed us to easily build rapport with the communities and collect data. The study site at Mirpur included three different socio-economic neighborhoods to increase variation of data. The study site at Fulbaria included two villages.

### Sampling

We selected our participants purposively in both sites to include persons from different settings, ages, socio-economic status, and occupational, educational and religious backgrounds according to the criteria described below for each qualitative method. Data saturation occurred after 16 in-depth interviews in urban Mirpur area and 18 in-depth interviews in rural Fulbaria with adult males and females. We identified three different socio-economic neighborhoods in urban Mirpur and recruiting participants from every tenth household. In each of the two selected villages in Fulbaria, we selected informants from every 10th household in purposively identified areas near four schools. We also selected two informants from Hindu families in each village.

We conducted eight focus group discussions in each of the study sites of Mirpur and Fulbaria. Each of these eight focus discussions took part with a pre-selected group, including some of the most influential members of Bangladeshi society, to maximize the variety of data collected relating to message dissemination for hygiene practices uptake. The groups included adult males, adult females, school boys, school girls, male teachers, female teachers, paramedics and religious leaders. Eight to 11 people participated in each focus group discussion. Selecting focus group discussion participants, to ensure that there was no overlap with the in-depth interview informants within the same geographic area, we started the selection process where the in-depth interview sampling had ended. We then enrolled adult male and female informants from the next 10th households. We prepared a list of all schools situated within or contiguous to both study sites and randomly selected two schools from each site; we selected male and female students of level three to five from one school and male and female teachers from the other. We selected paramedics from among those whom the informants commonly sought treatment as noted during in-depth interviews. To recruit religious leaders, we identified all of the mosques within the two study areas where Friday prayers take place.

For our observations, we identified the three closest public markets in each of our study sites. We chose 6 h during the busiest times of trading, between 7 AM to 10 AM in the morning and 5 PM to 8 PM in the evening to observe and record the behavior of shopkeepers and customers.

### Qualitative tools and data collection

We employed in-depth interviews and focus group discussions to explore community's perceptions, reported practices, and social norms related to respiratory infection and respiratory hygiene. Although the purpose of both of these qualitative tools overlapped, the focus group discussions covered a larger number of participants, and highlighted the vital role that each group could play in disseminating the messages. We used observations to explore and understand actual practice in natural settings. All three helped in triangulating our findings. A multidisciplinary team including public health specialists, social scientists, epidemiologists, and a communication specialist developed each of the qualitative guidelines for in-depth interview, focus group discussion and unstructured observation. Each tool was pretested in the field and revised (Appendix 1-3).

The data were collected by the first author, who is a sociologist, along with three anthropologists, who all had extensive experience in collecting qualitative data. Before data collection commenced we thoroughly reviewed the research objectives, research tools and specific data collection techniques to effectively and efficiently collect quality data. During in-depth interviews, lasting from 35 min to 60 min, and focus group discussions, ranging from 55 min to 90 min, we used an interview guide that started by asking people to list all of the illnesses they could think of that they associated with respiratory functions. We also asked about their perceptions on causes of respiratory illness, how respiratory infections are passed from one person to another, how disease transmission can be prevented, existing social norms, current practices related to respiratory hygiene, and for suggestions of messages and channels to promote respiratory hygiene behavior. We also conducted unstructured observations in markets to see respiratory hygiene practices in public places. During observation, we took notes about customers' and shopkeepers' behaviors linked to respiratory hygiene (Appendix 3). Nevertheless we did not count the events as we conducted unstructured observations in extremely crowded market places. Rather than quantify, we were more interested in observing people's actual respiratory hygiene practices including: whether people covered their mouths during coughing or sneezing; if so, how they covered their mouths (with hands/upper arm/cloth); whether they turned away from others while coughing or sneezing; whether they spat on the ground; and whether they washed their hands after nasal cleaning or coughing or sneezing into hands.

### Data analysis

We recorded the in-depth interviews and focus group discussions using audio recorders. We transcribed these audio recordings in Bengali, and to describe signs, symptoms and respiratory illness, we retained the local terminology. We manually coded the data according to our research objectives. After coding, we translated these data into English. We performed thematic content analysis in order to provide a descriptive of our result. Though we analyzed each in-depth interview and each focus group discussion separately, in the results we have drawn inferences collectively from both types of data. Moreover, in-depth interviews and focus group discussions findings were compared for consistency and were triangulated. No attempt was made to quantify study findings, as the number of informants interviewed was small. We followed the similar process for the unstructured observation notes.

### Ethical consideration and informed consent

Before taking part in the in-depth interviews and focus group discussions, we asked adult participants to provide written informed consent. We asked school students to provide assent with parental consent. This study protocol was reviewed and approved by ICDDR, B's Ethical Review Committee.

## Results

The majority (22/34) of our informants for in-depth interviews were aged 18-35 years; approximately 1 quarter were illiterate or could only sign their name. Over two-thirds (13/17) of female informants described themselves as homemakers (Table 1). In total, 144 participants took part in our focus group discussions. There were 70 adult males and 32 adult females, and 20 school boys and 22 school girls. Adult male focus group discussion participants were mostly street vendors or laborers in urban sites, and in rural sites most of them were farmers. In both study sites, adult female focus group discussion participants were homemakers.

**Table 1:** Socio-Demographic characteristics of the participants of in-depth interviews

Socio-Demographic Data	Mirpur		Fulbaria		Total (N = 34)
		
	Male (N = 8)	Female(N = 8)	Male (N = 9)	Female(N = 9)	
Age (years)					

18-35	4	6	5	7	22

36-50	3	1	3	2	9

>50	1	1	1	0	3

Education					

Illiterate/can sign	2	2	1	4	9

Class 1-5	1	4	1	3	9

Class 6-10	2	2	2	1	7

>Class 10	3	0	5	1	9

Occupation					

Laborer	2	2	0	1	5

Businessmen	3	0	3	0	6

Rickshaw/van puller	1	0	0	0	1

homemakers	0	6	0	7	13

Farmer	0	0	4	0	4

Others	2*	0	2	1	5

Settings and socio-economic status					

Kalabagan (slum area) in Mirpur	1	1	-	-	2

Baoniabad (lower class area)	5	5	-	-	10

Lalmatia-E (middle class area)	2	2	-	-	4

Chouder village	-	-	5	4	9

Andariapara village	-	-	4	5	9

Religion					

Islam	6	7	8	8	29

Hindu	2	1	1	1	5

*Tailor & private service

In the analysis of the 34 in-depth interviews, 16 focus group discussions, and six unstructured observations, there were no notable differences in the findings between the heterogeneous groups. We found similar responses and behaviors related to respiratory infections between urban and rural residents, different socio-economic groups, Muslims and Hindus, and school students and trained paramedics and lay persons. Therefore we have provided a summary of the findings aggregated across all study participants.

### Local terms of 'respiratory infection'

Our informants were not familiar with the Bengali translation of the term "respiratory infection" and did not have a specific Bengali word for this term. When asked to explain respiratory infection, informants used the phrase *'shas-proshassh jonito sangkromon'*, which literally translates as 'breathing and exhalation related infection'. The research team had to give detailed explanations and use hand gestures to indicate the focal areas of the body related to respiratory infection. When we asked informants to name some diseases transmitted during either breathing or exhalation, informants gave a wide variety of answers. Some answers had no direct relation to respiratory functions such as a stomach ache or skin diseases (Table 2).

**Table 2:** Signs, symptoms, and illnesses that informants associated with respiratory disease

Local terminology	Signs, Symptoms and illness
*Shash Kosto, Shash nite na para*	Difficulty breathing

*Joksha*	Tuberculosis

*Hapani, Adani*	Asthma

*Kup bhanga, shelesha*	Chest in-drawing

*Thanda laga*	Cold

*Hachi*	Sneeze

*Kashi*	Cough

*Pet betha*	Stomach ache

*Pet nama*	Cholera

*Pete oshukh, amashay*	Dysentery

*Matha betha*	Headache

*Ider chura*	Inflamed tonsils

*Shardi*	Runny nose

*Jor*	Fever

*Chok otha*	Conjunctivitis

*Boshonto, pansha*	Pox

*Mirki beram*	Epilepsy

*Kala-azar*	Kala-azar

*Khujli*	Skin diseases

### Perceived causes

Most of our informants associated transmission of respiratory infections with coming into close contact with a sick person's breath, cough droplets and spit; sharing a sick person's food or utensils; or allowing flies or mosquitoes that have had contact with human secreted substances (mucous/spit) to land on food. Focus group discussion data also supported these findings. A female teacher in a focus group discussion who was from the rural site stated;

"I need to avoid close contact with a sick person. My sister-in-law had difficulty breathing and her daughter-in-law took care of her and the daughter-in-law became infected with difficulty breathing."

Nevertheless, a few informants mentioned that respiratory infection could be contracted through blood transfusion, by genetic pre-disposition (heredity), by fate/luck and from contact with dead animals. A male teacher in a focus group discussion from the rural site attributed patterns of spread of infection to the direction the wind is blowing;

"If someone doesn't cover his mouth and nose during coughing or sneezing, the germs come out from his body. Anybody can be infected with these germs if they are carried in the same direction as the wind. How far away he is does not matter. But if the wind is blowing in the opposite direction other people will not be affected."

During in-depth interview, when we asked informants about how they caught cold during the previous 12 months, all of them linked catching a cold to imbalances of hot and cold, e.g. ambient temperatures or water temperatures. Specific responses included: exposure to excessive cold, change of weather from hot to cold, or vice versa; drinking cold water and bathing in cold water. A 36 year-old male informant from the urban site said;

"*Winter is going and summer is coming. It is hot during the day and cold at night and the mixing of hot and cold weather brings on a cold.*"

### Perceptions on preventive measures

Most informants reported that respiratory infections could be prevented by keeping distance from a sick person and by avoiding sharing utensils and food of a sick person. Paramedics also told us that when patients came to them with a respiratory infection they advised patients to keep warm and stay away from cold water. After probing, four informants also mentioned that washing their hands could protect them from respiratory illness. Nevertheless, these same informants continued to link the idea of internal imbalances of the body with catching a cold. One 43 year-old female informant from an urban site said;

*"A person should not be in contact with cold water for a long time to avoid getting cold/cough.*"

### Social norms and current practice

Informants from in-depth interviews and focus group discussions reported that there are no specific social norms related to respiratory hygiene. When we asked them about what they and their community considered as appropriate practices when someone has a cold in order not to spread it to other people, they mentioned a variety of actions that they felt were 'good behaviors'. These included covering the nose and mouth with hands or a handkerchief; turning the face away during sneezing or coughing; avoiding spitting, coughing, or sneezing into the environment; avoiding close contact with sick people; and keeping the body neat and clean. Religious leaders told us that hygiene was related to cleanliness before prayer time five times per day and that people should sneeze only into their left hand. In general they endorsed saying "P*raise be to Allah" (Alhamdulillah) *after sneezing. We found from our unstructured market observations that, almost universally, people did not practice these 'good behaviors' related to respiratory hygiene that they had identified during interviews and focus group discussions.

### Reported barriers to prevention

When exploring the perceived feasibility and effectiveness of handwashing to prevent respiratory infection, informants commonly stated that, if they did not wash their hands after coughing or sneezing into their hands, they could become ill. One adult informant from a rural site said;

"*Our hands are always dirty because of dust, which is everywhere. So without washing hands, anyone can be affected by any kind of respiratory disease.*"

Informants thought that it was not feasible or practical to wash hands after every event of coughing or sneezing, especially when someone has a runny nose. A 30 year old female informant from the urban site commented;

"*People don't wash their hands after sneezing and coughing. Is it possible to wash hands frequently? If you sneeze 100 times, will you wash your hands 100 times? But we should wash our hands before taking a meal.*"

### Messages and channels

After probing about how messages should be delivered and which channels of communication should be used, informants mentioned that awareness of respiratory hygiene behavior can be promoted by delivering messages related to their identified 'good behaviors, which could be disseminated through interpersonal communication, dramas, videos, television programs, during paramedic-patient interaction, and through the school curriculum. The teachers, imams and the paramedics told us that they can contribute to improving respiratory hygiene by disseminating related health messages. One religious leader in a focus group discussion from an urban site said;

*"We can deliver information among people during Friday prayers, the special prayer day for Muslims.*"

A female teacher in a focus group discussion from a rural site said;

"*We can deliver respiratory hygiene related disease messages among our students and at the time of stipend when all guardians come to the school and during mothers assembly we can discuss with parents about respiratory hygiene."*

## Discussion and Conclusions

Our findings suggest that local understandings about who gets sick, why, when, how illness spreads and how it can be prevented are varied, and therefore could contribute to the transmission of respiratory infections. Responses from our participants highlighted a contrast in people's minds between contracting a respiratory illness and its transmission and prevention. We have linked local interpretations of disease transmission to both the cultural model of hot-cold imbalance and the bio-medical understanding of transmission of respiratory infections. Understanding communities' perception of how an individual's behaviors can be linked to infectious disease transmission can help us engage in meaningful communication regarding behavior change. Well-designed and targeted communication interventions are more likely to be effective if they are theory-based or if they can be linked to a theoretical underpinning of established determinants of behavior and behavior change [14]. We could use these findings to develop culturally compelling behavior change interventions to interrupt respiratory disease transmission [15].

Informants related their individual experience of catching a cold with the hot-cold imbalance concept where health is maintained through equilibrium, or not having an internal imbalance in the body caused by an excess of either hot or cold. For example, keeping the body warm or avoiding cold elements can maintain internal equilibrium [16]. Our findings related to catching cold are similar to other anthropological and qualitative studies conducted on childhood acute respiratory infection during the 1990s. These studies suggested that people related the occurrence of acute respiratory infection to imbalances of hot and cold in the body, rather than to an infectious agent that can be transmitted from person to person [10,11,17-21]. For example, Bangladeshi mothers avoid feeding cold foods to prevent child from catching pneumonia [10], and Filipino mothers withhold breast milk from their infants after they have been exposed to cold weather, to prevent the child from catching a cold [21]. Identifying and understanding examples of these cultural perceptions could help in the development of communication messages to develop positive social norms related to respiratory hygiene behavior.

For transmission and prevention, most of the informants' responses aligned more closely to the bio-medical understandings of transmission that originated in the germ theory [22]. For example, although informants may not know that *Streptococcus pneumonae *and Influenza virus are some of the organisms responsible for pneumonia and influenza, and that this is where the common names of the illnesses were derived from, our informants were aware that respiratory infections were contagious and mentioned avoiding the cough, breath, spit and blood from an infected person. Communication messages could build on our informants' understanding of effective prevention measures.

About the prevention of respiratory infections, the community members had some perceptions that matched with the cultural model of hot-cold imbalance. Nevertheless, community members also had perceptions that aligned with the bio-medical concept as well. Though they perceived some 'good behaviors' were related to respiratory hygiene that could prevent respiratory infections, we observed that they did not translate those into practice. Informants told us that covering nose and mouth or turning the face away was a way to prevent the spread of infection. Nevertheless, almost all shopkeepers and customers we observed in the markets coughed into the air. In another study, where school children's respiratory hygiene behavior was observed, 956 (85%) of 1126 events, children coughed or sneezed into the air [9]. We speculate that the people we observed were not aware or did not value how their behavior could affect their health and that of others. Also many adults and young people do not have the habit of practicing respiratory hygiene, perhaps due to long-term exposure to an unhygienic physical and social environment. Communication messages that remind people to practice these 'good behaviors' on a daily basis could make positive changes in the overall environment.

A limitation of this study is that we collected data from only one urban site and one rural site. Nevertheless, the sites were typical of Bangladeshi communities, and we enrolled a heterogeneous mixture of informants from a variety of social groups. As our groups of informants had similar perceptions related to prevention and transmission, our findings from this formative study provide a foundation to design communication materials promoting respiratory hygiene practices that might be applicable for both rural and urban settings. Although we could not recruit informants from all geographic areas in the study sites for in-depth interviews and focus group discussions with adult males and females, we systematically selected participants from every 10th household in order to select them to diminish the bias of sample selection. Focus group discussions were limited in scope to fully explore complex beliefs. Nevertheless, we also conducted numbers of in-depth interviews, which were more appropriate to explore community beliefs. In focus group discussions, some participants were not vocal, and while the group facilitator tried to encourage them, this may have led to bias. Nonetheless, we triangulated our findings from in-depth interviews, focus group discussions and observations.

### Implications

Our understanding regarding the perceptions about why a person catches a cold and how respiratory infections are transmitted can be used to frame communication messages. Health professionals could use local terms to explain transmission of respiratory infections from the bio-medical perspective to highlight how changes in behavior could prevent transmission. These messages could make people more conscious about respiratory hygiene and then motivate them to follow their identified 'good behaviors' on a regular basis. Since the informants indicated that it is not feasible or practical to wash hands after every event of coughing and sneezing, our communication message could be to ask people to cough and sneeze into their upper arm or sleeve. We suggest piloting these messages with a variety of communication approaches and channels with all community members to reduce respiratory infections.

## Competing interests

The authors declare that they have no competing interests.

## Authors' contributions

SN developed the research protocol and all authors provided input in its design and methodological development. FAN developed the data collection instruments and supervises the data collection, analyzed and summarized the data. FAN drafted the initial manuscript and all authors commented on the drafts and approved the final manuscript. SL conceptualized the study and secured the funding. All authors have read and approved the final manuscript.

## Appendix 1: In-depth interview guideline

### Guide for in-depth interview

1. Please mention the name of any illness that you consider as respiratory. Is there any other local name for the illness?

2. Did you catch cold during the last 3/6/12 months? How do you think you were infected?

3. How is respiratory infection passed on from one person to another?

4. How can respiratory disease transmission be prevented?

5. Please tell me about the existing social norms of respiratory hygiene in your community? Which norms are common? Which norms do you think are important? *(probe for the perception of the respondent about the rationale for these norms)*

6. In your community, what are the current practices when people sneeze or cough?

7. Do you think there is any relationship between handwashing and respiratory diseases? Why or why not?

8. Have you received any health related information *(probe for respiratory illness)*? Where did you get it? Which sources/channel are credible to you and why? How often do you view these sources/channels?

9. How should the government communicate with people during an epidemic? *(Probe: by TV and radio, announcements after Friday prayers *etc.).

10. Has any of your health care providers ever given you any information about respiratory hygiene or advice on how respiratory infection can be prevented??

## Appendix 2: Focus group discussion guideline

### Guide for focus group discussion

Instruction: Every focus group discussion needs to be interactive within the study group. In focus group discussions it is common that certain participants dominate, while others keep quiet. We want to hear everyone's views, so try to encourage everyone to contribute. Before beginning, explain to the participants that there is no right or wrong answer and that you just want to learn from them and hear what they think. Begin with a round of introductions. Each participant should introduce him/herself.

1. Please mention the name of any illness that you consider as respiratory. Is there any other local name for the illness?

2. How respiratory infections pass on from one person to another?

3. How respiratory disease transmission can be prevented?

4. Please tell us something about the present norms, cultures and practices related to respiratory hygiene/diseases of your society.

5. In your community, what are the current practices when people sneeze or cough?

6. Do you think there is any relationship between handwashing and respiratory diseases? Why or why not?

7. What type of messages should be delivered to motivate people in your community to positively change their respiratory hygiene practices and what type of channel should be used to disseminate these messages?

8. How can you contribute to improve respiratory hygiene practices in your home and community

### Only for paramedics

9. Do you give any advice to your patients to prevent respiratory infection? If yes, what do you advise?

10. If you don't already give advice to patients, would you be interested to share any message that could prevent transmission of respiratory infection?

11. What type of message would you feels comfortable sharing with your patients?

12. Do you use any posters or pamphlets in your clinic for communicating health messages?

### Only for religious leaders

13. Is there any guidance or instruction given in Islam religion about hygiene in general or the transmission of respiratory illness or its prevention?

14. Do you give any other health messages in the community?

15. Would you be interested to share messages related to respiratory hygiene in the community?

## Appendix 3: Unstructured observation guideline

### Guide for market observation

(Observe and record all the activities related to respiratory hygiene)

• Whether anyone coughs or sneezes or cleans nasal secretions

• Covering of mouth with hands/upper arm/cloth while coughing or sneezing

• Using hands/cloths/handkerchief/other material for cleaning nasal secretions.

• Hand washing after coughing or sneezing into hands or cleaning nasal secretions

• Spitting cough or spit, and where.

• Turning away from others while coughing or sneezing or cleaning nasal secretions

• Widening distances from nearby people when someone coughs or sneezes or cleans nasal secretions.

•Widening distances when someone around them coughs/sneezes/spits/cleans nasal secretions.

## Pre-publication history

The pre-publication history for this paper can be accessed here:

http://www.biomedcentral.com/1471-2458/11/901/prepub
